# Combined Prognostic Value of Preprocedural Protein–Energy Wasting and Inflammation Status for Amputation and/or Mortality after Lower-Extremity Revascularization in Hemodialysis Patients with Peripheral Arterial Disease

**DOI:** 10.3390/jcm13010126

**Published:** 2023-12-25

**Authors:** Yoshitaka Kumada, Norikazu Kawai, Narihiro Ishida, Yasuhito Nakamura, Hiroshi Takahashi, Satoru Ohshima, Ryuta Ito, Hideo Izawa, Toyoaki Murohara, Hideki Ishii

**Affiliations:** 1 Department of Cardiovascular Surgery, Matsunami General Hospital, Kasamatsu 501-6062, Japan; ykumada1206@gmail.com (Y.K.); norikazu.kawai@gmail.com (N.K.); alpineskierjp@aol.com (N.I.); o2001070@yahoo.co.jp (Y.N.); 2 Department of Cardiology, Fujita Health University School of Medicine, Toyoake 470-1192, Japan; hirotaka@fujita-hu.ac.jp (H.T.); izawa@fujita-hu.ac.jp (H.I.); 3 Department of Cardiology, Nagoya Kyoritsu Hospital, Nagoya 454-0933, Japan; kaku@nagoya-pet.or.jp (S.O.); ryu-i@msi.biglobe.ne.jp (R.I.); 4 Department of Cardiology, Nagoya University Graduate School of Medicine, Nagoya 466-8550, Japan; murohara@med.nagoya-u.ac.jp; 5 Department of Cardiovascular Medicine, Gunma University Graduate School of Medicine, 3-39-22 Showa-machi, Maebashi 371-8511, Japan

**Keywords:** lower-extremity revascularization, peripheral artery disease, hemodialysis, geriatric nutritional risk index, C-reactive protein

## Abstract

Protein–energy wasting is associated with inflammation and advanced atherosclerosis in hemodialysis patients. We enrolled 800 patients who had undergone successful lower-extremity revascularization, and we investigated the association among the Geriatric Nutritional Risk Index (GNRI) as a surrogate marker of protein–energy wasting, C-reactive protein (CRP), and their joint roles in predicting amputation and mortality. They were divided into lower, middle, and upper tertiles (T1, T2, and T3) according to GNRI and CRP levels, respectively. Regarding the results, the amputation-free survival rates over 8 years were 47.0%, 56.9%, and 69.5% in T1, T2, and T3 of the GNRI and 65.8%, 58.7%, and 33.2% for T1, T2, and T3 of CRP, respectively (*p* < 0.0001 for both). A reduced GNRI [adjusted hazard ratio (aHR) 1.78, 95% confidence interval (CI) 1.24–2.59, *p* = 0.0016 for T1 vs. T3] and elevated CRP (aHR 1.86, 95% CI 1.30–2.70, *p* = 0.0007 for T3 vs. T1) independently predicted amputation and/or mortality. When the two variables were combined, the risk was 3.77-fold higher (95% CI 1.97–7.69, *p* < 0.0001) in patients who occupied both T1 of the GNRI and T3 of CRP than in those who occupied both T3 of the GNRI and T1 of CRP. In conclusion, patients with preprocedurally decreased GNRI and elevated CRP levels frequently experienced amputation and mortality, and a combination of these two variables could more accurately stratify the risk.

## 1. Introduction

Recently, the prevalence of chronic kidney disease (CKD) has been significantly increasing [1,2,3]. Renal impairment is associated with a high incidence of cardiovascular disease [4,5,6]. Thus, cardio-renal interaction has received attention. In particular, it has been reported that end-stage CKD patients requiring maintenance hemodialysis (HD) therapy are recognized as the highest-risk population for cardiovascular disease, including peripheral artery disease (PAD) [7,8,9]. Lower-limb revascularization such as bypass surgery or endovascular therapy (EVT) has been commonly performed to treat PAD. However, poorer prognoses, such as higher amputation or mortality rates, remain a major clinical problem in patients with advanced CKD after revascularization, regardless of whether it was bypass surgery [10,11] or EVT [12,13], compared to those without. Unfortunately, the dismal outcomes have not been improved over the last decade despite improvements in the medical management of HD patients over the same period [14,15,16,17]. In such a situation, simple risk stratification to predict future outcomes may be clinically important in patients on HD.

On the other hand, nutritional status is one of the key points in patients with CKD. In clinical settings, protein–energy wasting (PEW) [18,19,20], a state of decreased body protein mass and energy fuel, is commonly seen in patients with CKD [21,22]. PEW can result from a poor diet as well as inflammatory processes [23,24], and inflammatory status itself is associated with higher cardiovascular and all-cause mortality in this population [25,26]. Moreover, we previously reported that the presence of PEW and inflammatory status was independently associated with a reduced ankle–brachial index (ABI) and that patients with these factors had poorer survival rates than those without [27]. Therefore, in this study, we investigated the association of preprocedural Geriatric Nutritional Risk Index (GNRI) values [28,29], which may be a surrogate marker of PEW, and C-reactive protein (CRP) levels with limb amputation and/or mortality after lower-extremity revascularization in patients with CKD undergoing HD.

## 2. Methods

### 2.1. Patients

This was a retrospective study. From January 2009 to April 2018, a total of 800 consecutive HD patients who underwent successful lower-extremity revascularization (535 undergoing EVT and 265 undergoing bypass surgery) after the measurement of preprocedural GNRI and CRP levels at Matsunami General Hospital (Kasamatsu, Japan) and Nagoya Kyoritsu Hospital (Nagoya, Japan) were enrolled in this study. Patients with acute limb ischemia were excluded in advance.

Clinical information including patients’ characteristics and established risk factors, indications for revascularization, and target lesions for PAD was obtained from medical records. Briefly, in all patients undergoing EVT, iliac and femoropopliteal lesions were expanded with an ordinary balloon at first. A stent was implanted if there was a residual stenosis with a luminal diameter >30% and/or a residual flow-limiting dissection. In contrast, no stent was used in infrapopliteal lesions, even if residual stenosis or dissection was observed after balloon angioplasty. As for bypass surgery, we chose the ipsilateral or contralateral great saphenous vein as the graft. The operation was performed under general anesthesia.

The study was conducted according to the guidelines of the Declaration of Helsinki and approved by the Ethics Committees of Matsunami General Hospital (code: 573) and Nagoya Kyoritsu Hospital (code: K132-02), respectively. The need to obtain written informed consent and provide information regarding how to opt out of this study on the website of each hospital was waived due to the retrospective nature of the study.

### 2.2. GNRI and CRP Measurements

Blood samples were collected before the day of the procedure to measure serum albumin and CRP levels. We calculated the GNRI from individually obtained serum albumin levels and each patient’s height and body weight [30]:

GNRI = [14.89 × albumin (g/dL)] + [41.7 × (body weight/ideal body weight)]

The body weight/ideal body weight ratio was set to one when the patient’s body weight exceeded the ideal body weight. Ideal body weight was defined as the value calculated from the patient’s actual height and a body mass index of 22 [30]. Enrolled patients received HD therapy one day prior to the procedure, and body weight after HD therapy was checked to calculate the GNRI. Serum CRP levels were measured using a latex-enhanced, highly sensitive CRP immunoassay. Then, according to GNRI and CRP levels, enrolled subjects were divided into tertiles, respectively.

### 2.3. Follow-Up

We routinely followed up the enrolled patients after discharge at 1, 3, and 6 months during the first year. Thereafter, we followed up them at yearly intervals and additionally performed duplex scanning to check for lower-limb ischemia. If we could not conduct a hospital follow-up, the patient was interviewed over the telephone if possible, and the follow-up ended on the day of the last visit if we could not confirm the status of the patient. The follow-up period ended in January 2019. The primary outcome was amputation-free survival (AFS), officially defined as freedom from above-ankle amputation of the index limb or death from any cause [31].

### 2.4. Statistical Analyses

All statistical analyses were performed using SPSS version 21 (IBM Corp., Armonk, NY, USA).

Normally distributed variables were expressed as the mean ± SD, and asymmetrically distributed data were given as the median and interquartile range. Differences among the groups were evaluated using one-way analysis of variance or the Kruskal–Wallis test for continuous variables and the chi-square test for categorical variables. Using the Kaplan–Meier method, the AFS rates of the groups were expressed. In addition, a log-rank test was used to compare the differences. Hazard ratios (HRs) and 95% confidence intervals (CIs) were calculated for each factor using Cox proportional hazards models. To identify factors independently predicting the outcome, we entered all baseline variables with *p* < 0.05 in a univariable analysis into a multivariate model. To clarify whether the predictability of amputation and/or mortality could improve after the addition of the GNRI alone, CRP alone, and both into a baseline model with established risk factors, the C-index, net reclassification improvement (NRI), and integrated discrimination improvement (IDI) were calculated. The C-index, which is defined as the area under receiver operating characteristic curve between the individual predicted probabilities of the endpoints and the incidence of the endpoints, was compared among all the predictive models [32]. NRI estimates were used to quantify how much better one model predicted the outcome compared to another without the variable of interest [33]. Differences were defined to be statistically significant at a two-sided *p* value less than 0.05.

## 3. Results

### 3.1. Patient Characteristics

**Figure 1:**
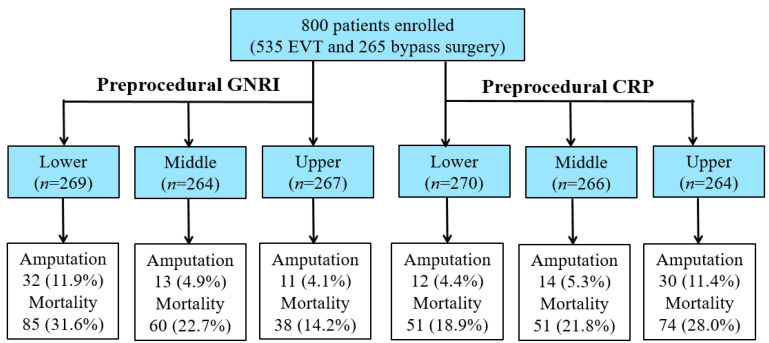
Study design and events.

Patients were divided into tertiles according to GNRI levels, respectively (tertile 1 (T1): <88.1; T2: 88.1–96.7; T3: >96.7), and CRP levels (T1: <2.0 mg/L; T2: 2.0–12.6 mg/L; T3: >12.6 mg/L) (Figure 1).

The enrolled patients’ characteristics are shown in Table 1 and Table 2. Those with lower GNRI values had higher CRP levels [11.3 (2.9–44.5) mg/L, 4.0 (1.0–14.0) mg/L, and 3.0 (1.0–12.0) mg/L in T1, T2, and T3, respectively; *p* < 0.0001] and a higher prevalence of ulcer/gangrene (49.6%, 44.6%, and 27.7% in T1, T2, and T3, respectively; *p* < 0.0001). Similarly, those with higher CRP also had lower GNRI values (94.3 ± 9.4, 93.1 ± 9.7, and 89.1 ± 10.1 in T1, T2, and T3, respectively; *p* < 0.0001) and higher prevalence of ulcer/gangrene (23.5%, 34.5%, and 63.9% in T1, T2, and T3, respectively; *p* < 0.0001).

### 3.2. Predictive Value of the GNRI and CRP

**Figure 2:**
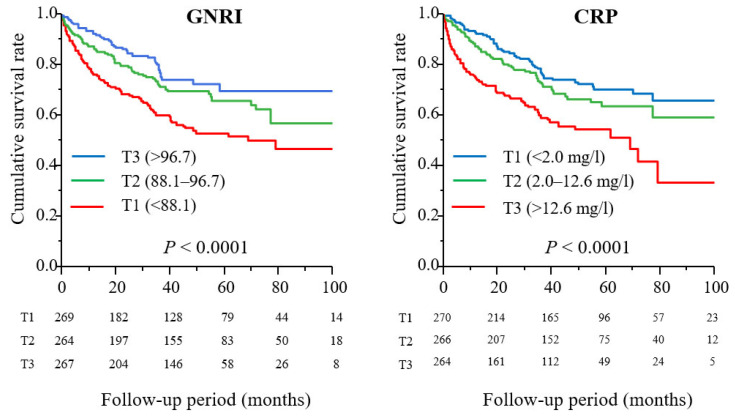
Amputation-free survival rates in tertiles of GNRI (left panel) and CRP (right panel).

A total of 56 (7.0%) patients required major amputation during the follow-up period (median, 43 months), and 183 (22.9%) patients died. Kaplan-Meier analysis showed that the AFS rates for 8 years were 47.0%, 56.9%, and 69.5% in T1, T2, and T3 of the GNRI and 65.8%, 58.7%, and 33.2% in T1, T2, and T3 of CRP, respectively (*p* < 0.0001 for both) (Figure 2).

After adjustment for male sex, age, previous coronary artery disease, procedure (EVT vs. bypass surgery), below-the-knee artery disease, and ulcer/gangrene as covariates with *p* < 0.05 in a univariate analysis, a decreased GNRI [adjusted HR 1.78, 95% CI 1.24–2.59, *p* = 0.0016 for T1 vs. T3] and elevated CRP (adjusted HR 1.86, 95% CI 1.30–2.70, *p* = 0.0007 for T3 vs. T1) were identified as independent predictors of amputation and/or mortality (Table 3). Similar results were obtained for the amputation and mortality rates.

### 3.3. Combined Predictive Value of the GNRI and CRP

**Figure 3:**
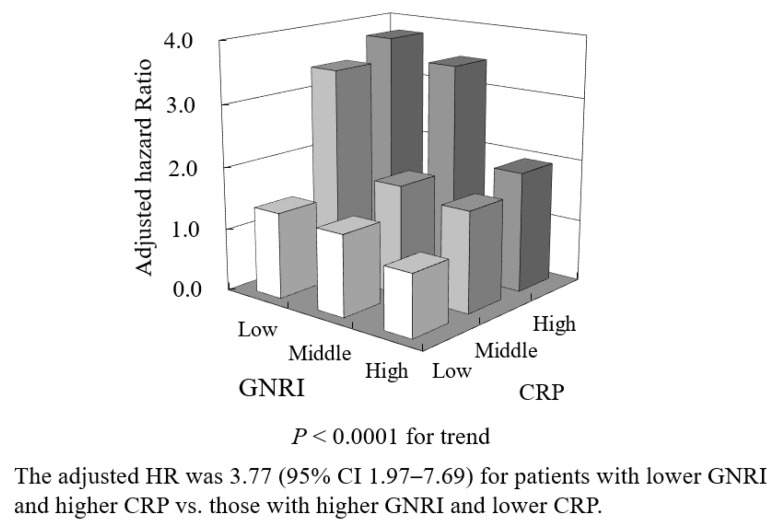
Adjusted hazard ratio (HR) for amputation and/or mortality in combinations of tertiles of GNRI and CRP.

The combination of the two variables could stratify the risk of amputation and/or mortality, and the risk was 3.77-fold higher (95% CI 1.97–7.69, *p* < 0.0001) in patients occupying GNRI T1 and CRP T3 than in those occupying GNRI T3 and CRP T1 (Figure 3).

**Figure 4:**
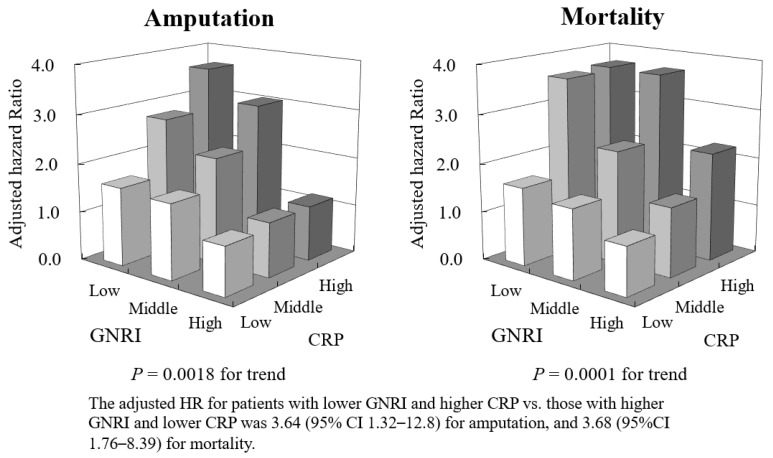
Adjusted hazard ratio (HR) for amputation (left panel) and mortality (right panel) in combinations of tertiles of GNRI and CRP.

Similar results were also obtained for amputation and mortality (adjusted HR 3.64, 95% CI 1.32–12.8, *p* = 0.0018 for amputation and adjusted HR 3.68, 95% CI 1.76–8.39, *p* < 0.0001 for mortality for GNRI T1 with CRP T3 vs. GNRI T3 with CRP T1, respectively) (Figure 4).

For model discrimination, the addition of both the GNRI and CRP to a predicting model with established risk factors improved the C-index (from 0.661 to 0.716, *p* = 0.0021), NRI (0.508, *p* < 0.0001), and IDI (0.042, *p* < 0.0001). They were even greater than those of either individual variable (NRI 0.145, *p* = 0.047 and IDI 0.006, *p* = 0.035 vs. the GNRI alone and NRI 0.427, *p* < 0.0001 and IDI 0.029, *p* < 0.0001 vs. CRP alone, respectively) (Table 4). The measurement of both PEW and CRP can more accurately stratify risk in hemodialysis patients with PAD who undergo EVT.

## 4. Discussion

Our results clearly demonstrated that a preprocedural decline in the GNRI and an elevated CRP level, which might reflect PEW and chronic inflammation status, resulted in poor AFS after lower-limb revascularization in patients undergoing HD and that the combination of the two variables could more accurately stratify the risk of poor AFS and could provide significantly better predictive performance than either variable alone. Because a simple method for risk stratification in such a high-risk population is attractive, our findings might be of significance in clinical practice.

Numerous studies have reported consistently poorer prognosis after lower-limb revascularization in patients undergoing HD than in the general population in spite of advances in the medical management of HD [10,11,12,13,14,15,16,17]. In previous studies, we reported the following findings: (1) Severe/moderate nutritional risk (GNRI < 92) was higher in patients undergoing HD (53%) than in the elderly general population (21–43%) despite HD patients (average of 64 years) being younger than the elderly general population (80–85 years) [22]. (2) In patients who underwent bypass surgery, preprocedural CRP levels were markedly higher in HD patients than in non-HD patients (median of 11 mg/L vs. 4 mg/L) [34]. (3) Interestingly, preprocedural elevated CRP levels could predict poor AFS only in HD patients and not non-HD patients who underwent infrapopliteal bypass surgery [34]. Thus, our findings in the present study might be reasonably explained, and PEW and chronic inflammation status, a CKD-specific morbidity, might be considered to be one of the causes of poor AFS after lower-limb revascularization in HD population.

In addition, we previously reported that the limb salvage rate after bypass surgery was comparable between HD and non-HD patients when performing propensity score matching with unfavorable factors, including preprocedural CRP levels [35]. This fact suggests the possibility of improved prognosis if inflammation status is adequately managed, even in patients undergoing HD. In this context, the recently developed wound, ischemia, and foot infection (WIfI) scoring system is considered important for assessing the risk of poor AFS [36]. Unfortunately, WIfI scores were not measured in the present study. The association among variables included in WIfI scores and prognosis in such a high-risk population should be clarified in the near future.

The condition of PEW was previously referred to as malnutrition, inflammation, and atherosclerosis (MIA) syndrome before it was officially defined by the International Society of Renal Nutrition and Metabolism (ISRNM) [23,24]. We have previously reported the close association of both a decreased GNRI and elevated CRP with an abnormal ABI [27]. An abnormal ABI also reportedly reflects not only PAD but also systemic atherosclerosis [37,38]; thus, the previous findings might manifest as MIA syndrome. In this context, patients with decreased preprocedural GNRI values and elevated CRP levels were considered to have advanced atherosclerosis and poor prognosis in the present study. Thus, physicians should pay more attention to these unfavorable conditions in those with malnutrition and elevated inflammatory status.

Finally, the addition of both preprocedural GNRI and CRP levels to a predictive model with established risk factors such as age, infrapopliteal disease, and ulcer/gangrene significantly improved the predictability of poor AFS after revascularization to a greater extent than the addition of the GNRI or CRP alone. Thus, measurement of both variables before procedures might be clinically beneficial for predicting prognosis more accurately because these variables are also easily obtained in daily practice.

The present study has several limitations. First, it was a non-randomized, retrospective study. Second, all the study participants were Japanese, a group that reportedly has a lower atherosclerotic risk than patients in the United States and Europe [39]. Third, the study participants were from two centers only. Fourth, once again, we could not assess the WIfI scores. The lack of data regarding wound or infection status in the limbs might be the most important limitation of the study. Last, there were no precise data on medications. These limitations should be considered when interpreting our results.

## 5. Conclusions

Although lower-extremity revascularization is commonly performed in hemodialysis patients, poor prognosis remains a major problem. In our study, a preprocedural decline in the GNRI and an elevated CRP level, which reflect PEW and chronic inflammation status, are closely associated with poor AFS after lower-limb revascularization in chronic HD patients. Furthermore, the combination of the two variables could not only stratify the risk of amputation and/or mortality but also improve predictive performance when added to established risk factors. Our findings might easily stratify clinical outcomes in HD population at high risk.

## Figures and Tables

**Table 1:** Patient clinical characteristics depending on GNRI levels.

		GNRI			
	All Patients (*n* = 800)	<88.1 (*n* = 269)	88.1–96.7 (*n* = 264)	>96.7 (*n* = 267)	*p* Value
Male gender (%)	66.9	62.5	67.4	70.7	0.12
Age (years)	67 ± 10	69 ± 10	67 ± 9	66 ± 10	0.0024
Diabetes (%)	63.3	63.2	64.7	61.8	0.78
Hypertension (%)	62.1	58.0	61.7	66.7	0.12
Dyslipidemia (%)	24.5	18.2	25.8	29.6	0.0076
Smoking (%)	25.7	18.6	31.4	27.2	0.0078
Body mass index (kg/m^2^)	21.2 ± 3.3	19.2 ± 2.9	21.0 ± 2.6	23.3 ± 3.0	<0.0001
Coronary artery disease (%)	63.5	58.7	63.9	67.8	0.092
Stroke (%)	16.9	18.6	15.5	16.5	0.63
Indications (%)					<0.0001
Claudication	47.1	36.8	43.9	60.4	
Rest pain	12.3	13.6	11.5	11.9	
Ulcer/gangrene	40.6	49.6	44.6	27.7	
GNRI	92.0 ± 9.8	81.4 ± 5.8	92.3 ± 2.4	102.4 ± 5.3	<0.0001
CRP (mg/L)	5.1 (2.0–20.0)	11.3 (2.9–44.5)	4.0 (1.0–14.0)	3.0 (1.0–12.0)	<0.0001
Preprocedural ABI	0.62 (0.45–0.79)	0.65 (0.41–0.87)	0.57 (0.44–0.79)	0.64 (0.49–0.77)	0.35
Procedure (%)					<0.0001
Bypass surgery	33.1	38.7	39.8	21.0	
Endovascular therapy	66.9	61.3	60.2	79.0	
Number of lesions	825	282	271	272	
Target artery (%)					<0.0001
Iliac	18.1	22.7	17.6	14.3	
Femoropopliteal	62.1	52.8	59.1	72.8	
Below-knee	21.3	24.5	23.3	12.9	

GNRI, Geriatric Nutritional Risk Index; CRP, C-reactive protein; ABI, ankle–brachial index.

**Table 2:** Patient clinical characteristics depending on serum CRP levels.

	Serum CRP			
	<2.0 mg/L (*n* = 270)	2.0–12.6 mg/L (*n* = 266)	>12.6 mg/L (*n* = 264)	*p* Value
Male gender (%)	62.6	69.2	68.9	0.19
Age (years)	66 ± 10	67 ± 10	69 ± 10	0.046
Diabetes (%)	60.4	60.9	63.3	0.091
Hypertension (%)	63.7	61.7	61.0	0.80
Dyslipidemia (%)	25.9	24.8	22.7	0.68
Smoking (%)	27.5	24.8	24.9	0.75
Body mass index (kg/m^2^)	20.9 ± 3.1	21.2 ± 3.0	21.5 ± 3.7	0.15
Coronary artery disease (%)	63.2	65.0	63.5	0.78
Stroke (%)	18.6	18.4	13.6	0.22
Indications (%)				<0.0001
Claudication	61.9	49.6	29.6	
Rest pain	14.6	15.9	6.5	
Ulcer/gangrene	23.5	34.5	63.9	
GNRI	94.3 ± 9.4	93.1 ± 9.7	89.1 ± 10.1	<0.0001
CRP (mg/L)	1.0 (1.0–2.0)	5.9 (3.9–8.0)	39.5 (20.0–70.0)	<0.0001
Preprocedural ABI	0.65 (0.47–0.79)	0.63 (0.44–0.82)	0.57 (0.43–0.76)	0.23
Procedure (%)				<0.0001
Bypass surgery	22.2	30.5	47.0	
Endovascular therapy	77.8	69.5	53.0	
Number of lesions	274	271	280	
Target artery (%)				<0.0001
Iliac	22.3	18.5	13.6	
Femoropopliteal	69.7	64.6	52.1	
Below-knee	8.0	17.0	34.3	

GNRI, Geriatric Nutritional Risk Index; CRP, C-reactive protein; ABI, ankle–brachial index.

**Table 3:** Predictive value of GNRI and CRP for amputation and mortality.

	Non-Adjusted		Adjusted **	
	HR (95% CI)	*p* Value	HR (95% CI)	*p* Value
Amputation or death				
GNRI (vs. T3)		<0.0001 *		0.0070 *
T2	1.46 (1.03–2.09)	0.031	1.42 (0.97–2.09)	0.070
T1	2.18 (1.57–3.07)	<0.0001	1.78 (1.24–2.59)	0.0016
CRP (vs. T1)		<0.0001 *		0.0026 *
T2	1.32 (0.93–1.89)	0.11	130 (0.90–1.91)	0.15
T3	2.33 (1.67–3.27)	<0.0001	1.86 (1.30–2.70)	0.0007
Amputation				
GNRI (vs. T3)		<0.0001 *		0.032 *
T2	1.11 (0.78–2.44)	0.79	1.05 (0.46–2.39)	0.89
T1	3.17 (1.70–6.37)	0.0002	2.01 (1.04–4.12)	0.034
CRP (vs. T1)		0.0003 *		0.045 *
T2	1.26 (0.58–2.79)	0.54	1.01 (0.45–2.23)	0.98
T3	3.35 (1.75–6.85)	0.0001	2.02 (1.02–4.25)	0.042
Mortality				
GNRI (vs. T3)		0.0002 *		0.0083 *
T2	1.51 (1.03–2.23)	0.032	1.51 (0.99–2.33)	0.052
T1	2.12 (1.48–3.09)	<0.0001	1.87 (1.25–2.84)	0.0020
CRP (vs. T1)		0.0004 *		0.043 *
T2	1.30 (0.89–1.90)	0.17	1.29 (0.86–1.94)	0.20
T3	2.03 (1.42–2.93)	<0.0001	1.64 (1.11–2.45)	0.012

*: *p* for trend. **: adjusted for male sex, age, previous coronary artery disease, endovascular therapy (vs. bypass surgery), below-knee artery disease, and ulcer/gangrene as covariates with *p* < 0.05 in a univariate analysis.

**Table 4:** Discrimination performance of each prediction model for amputation or mortality using the C-index, net reclassification improvement (NRI) and integrated discrimination improvement (IDI).

	C-Index (95% CI)	*p* Value	NRI	*p* Value	IDI	*p* Value
Established risk factors *	0.661	reference		reference		reference
+GNRI	0.710	0.0060	0.456	<0.0001	0.037	<0.0001
+CRP	0.681	0.0034	0.217	0.0063	0.014	0.0001
+GNRI and CRP	0.716	0.0021	0.508	<0.0001	0.042	<0.0001
+GNRI and CRP vs. +GNRI	0.006 **	0.047	0.145	0.047	0.006	0.035
+GNRI and CRP vs. +CRP	0.035 **	0.038	0.427	<0.0001	0.029	<0.0001

*: model includes male sex, age, previous coronary artery disease, endovascular therapy (vs. bypass surgery), below-knee artery disease, and ulcer/gangrene. **: estimated difference.

## Data Availability

The data presented in this study are available on request from the corresponding author.
